# Excision of pediatric craniopharyngioma with complicated features: the value of additional "tailored" cavernous sinus exposure in the standard pterional approach

**DOI:** 10.3171/2026.7.FOCVID25239

**Published:** 2026-10-01

**Authors:** Kuntal Kanti Das, Chandana Kanuru, Shreyash Rai, Neha Nigam, Kamlesh Singh Bhaisora, Arun Kumar Srivastava

**Affiliations:** 1 Department of Neurosurgery, Sanjay Gandhi Postgraduate Institute, Lucknow, Uttar Pradesh; and; 2 Department of Pathology, Sanjay Gandhi Postgraduate Institute, Lucknow, Uttar Pradesh, India

**Keywords:** retrochiasmatic craniopharyngioma, transcavernous, pterional, excision, calcification

## Abstract

Excision of craniopharyngioma (CP) can be challenging. Pediatric CPs pose the unique challenge of ensuring a satisfactory onco-functional balance while navigating the limited therapeutic options in this age group. Here the authors present the value of "tailored" cavernous sinus exposure and neurovascular mobilization to minimize the inevitable and, at times, potentially harmful manipulation of the optic and oculomotor nerves associated with an anterolateral transcranial approach. This additional surgical step also ensures an inferior-to-superior surgical trajectory through a caudal durotomy, significantly improving the tumor-hypothalamus interface visualization, which is otherwise a limitation of the pterional approach.

The video can be found here: https://stream.cadmore.media/r10.3171/2026.7.FOCVID25239

## Transcript

In this video, the authors present "Excision of pediatric craniopharyngioma with complicated features: the value of additional ‘tailored’ cavernous sinus exposure in the standard pterional approach."

### 0:31 Disease Background: Craniopharyngioma.

Craniopharyngioma remains a daunting neurosurgical challenge,^1^ and this is one of the most complex suprasellar tumors. The tumor location and the neurovascular relations often result in suboptimal tumor resection rates, and postoperative morbidity could be substantial. Multicompartmental tumor growth, heavy tumoral calcification, a retrochiasmatic extension, a previous surgery, a posterior fossa extension,^2,3^ etc., have been thought to be complicating features in craniopharyngiomas in previous publications. The pediatric craniopharyngiomas are often large. They present with headaches and have higher incidence of postoperative endocrine dysfunction.^4,5^ The large chunks of calcification and tight hypothalamic adherence require special surgical consideration, and, in this video, the authors present the utility of an additional extradural cavernous sinus exposure, anterior clinoidectomy, and neurovascular mobilization^6^ in expanding the surgical corridor and enhanced hypothalamic visualization in CPs with complicated features.

### 1:22 Case Details.

The case in point is a 3-year-old girl who presented with headache and visual complaints for 6 months, without any other neurological deficits. On neurological examination, the visual acuity was 6/12 on the right side, 6/18 on the left. There was bilateral papilledema. The visual field could not be reliably tested. There were no other neurological deficits, and the endocrine profile in the preoperative period was normal.

### 1:42 Preoperative Imaging.

Here we can see the preoperative imaging. On the upper panel, we can see the CT scans, which show heavy calcification in the central area and cystic component going superiorly toward the third ventricle. On MR images, we can see that the tumor has a cystic component, which has extended posteriorly to the prepontine area. Superiorly, it has extended to the third ventricular area, and the calcification is appearing as dark, and it is located in the center, just in the suprasellar space.

### 2:09 Operative Plan.

This patient was planned for a surgical resection using a right pterional transcavernous approach.

### 2:14 Rationale for the Surgical Approach.

The rationale for this additional modification to the pterional approach was that the pterional approach has the limitation of excessive manipulation of the oculomotor and the optic nerves in order to access the suprasellar space. The proposed modification would be helpful in mobilizing the optic and oculomotor nerves so that it can withstand the handling and manipulation, expansion of the conventional corridors to deal with the larger calcified chunks, minimization of brain handling by dint of a caudal dural opening, and an inferior to superior lookup view, which can somehow compensate for the difficulty in dissecting the tumor-hypothalamic interface, which can be a limitation of the traditional pterional approach.

### 2:53 Position and Incision.

The patient was placed supine with head rotation and slight extension. A reverse question mark as shown here was used. An interfacial dissection and zygoma lowering helped us reach the base of the temporal fossa, and a frontotemporal craniotomy was raised.

### 3:07 Craniotomy, Extradural Exposure of the Anterior Clinoid Process, and Optic Canal Unroofing.

Here we can see a right pterional craniotomy has been done. Here we see that the superior orbital fissure is being decompressed after a small posterior orbitotomy. The meningo-orbital band is divided, and the frontotemporal dural fold peeling is started. As this peeling proceeds, we can see that the anterior clinoid process progressively gets exposed and more superficial. Here we can see the optic canal. The optic canal is unroofed with the help of drill and upcutting rongeurs. We can see that the clinoid has already started moving. Here we release some amount of CSF from around the perioptic cisterns, the chiasmatic cistern. It makes the brain lax.^7^

### 4:02 Peeling of the Lateral Cavernous Sinus Wall Dural Duplication and Completion of Extradural Exposure.

The peeling is continued posteriorly. Here we can see the V2 division of the trigeminal nerve. The dural duplication over it is divided. As we move posteriorly and divide the middle meningeal artery, we expose the V3 segment. The foramen of ovale was expanded. The peeling is continued posteriorly. As we keep on peeling, we expose the Meckel’s cave, which is shown here. Additional CSF is released from the Meckel’s cave area. The clinoid process is then completely removed by drilling the optic strut. This exposes the clinoidal cone. The structures on the lateral wall of the cavernous sinus as well as the second nerve are shown. You can see the fourth cranial nerve, the third cranial nerve.

### 4:53 Durotomy and Corridor Expansion.

The dura is now opened horizontally, parallel to the base so that we expose minimum part of the cortex. Here we see that the optic nerve has been stretched by the suprasellar tumor. The sheath of the optic nerve is divided to make the optic nerve lax. Additionally, we also divide the distal dural ring so that we have a caudal exposure near the carotid. The retrocarotid space is also seen. Here we can see that the tumor is expanding posteriorly as well. We can see the third cranial nerve.

### 5:23 Tumor Resection: Delivery of the Heavy Calcified Part.

We see that the tumor is projecting through the interoptic space as well. This is the heavy calcified part. It is very difficult to be broken with scissors and dissector. But the good thing was, it was covered by soft areas, which allowed intermittent debulking through the expanded corridors. Here we can see that the carotico-optic space is being used. As the tumor is progressively debulked and freed from the adjoining areas with the help of the dissector. With the tumor decompressed, gentle teasing movements can deliver the tumor out from the Liliequist membrane, which is a natural protector of the brainstem and the basilar bifurcation area. With gentle dissection, we are able to take the tumor out from the widened interoptic space.

### 6:10 Tumor Resection: Remaining Soft Suprasellar Part.

Now, using the cross-corridor technique, we enter into the tumor. Here we are mobilizing the rest of the soft part of the tumors through carotico-optic space. The tumor is pushed into the interoptic space, and we do the further dissection from here. Progressively, the softer portion of the tumor is removed. Now the tumor is being removed from multiple corridors. This became feasible because of the neurovascular mobilization. We can see the superior pole of the tumor from an inferior to superior lookup view. Posteriorly, we can see that the tumor has been pushed anteriorly into the carotico-optic space. The calcified wall of the tumor is being removed.

### 7:10 Tumor Resection: Separation From the Flattened Pituitary Stalk.

The pituitary stalk has been compressed and flattened here. With progressive dissection and piecemeal removal, now we can see the compressed pituitary stalk.

### 7:22 Tumor Resection: Posterior Cranial Fossa Part.

Here we can see the third cranial nerve. The cisternal segment of the third cranial nerve is mobilized. It allows visualization of the prepontine space. We can see the basilar artery here. The tumor, which is located close to the basilar artery, is removed from the carotico-optic space. The tumor is being removed from behind the carotid bifurcation.

### 7:52 Tumor Resection: Hypothalamic Visualization.

Superiorly, we can see the beautiful view of the hypothalamus. This inferior to superior lookup view is extremely essential to directly visualize the hypothalamus. Small tumor remnant which was stuck to the hypothalamus here was left behind. The handled internal carotid artery was covered with papaverine-soaked Gelfoam.

### 8:15 Postoperative Radiological Outcome.

Postoperative scan, we see near-total resection of the tumor, and the follow-up MRI at 18 months shows that the tumor has not regrown and the disease is stable.

### 8:25 Postoperative Clinical Outcome.

Clinically, the patient had stable postoperative vision with slight improvement in the postoperative period. She was discharged on postoperative day 12 after stabilization of the diabetes insipidus. In the follow-up, she developed a permanent diabetes insipidus, and she required replacement dosage of thyroxine, corticosteroids, and desmopressin. However, there were no other features of hypothalamic dysfunction in the follow-up. At 2 years, she has shown no recurrence of the tumor, she is under regular follow-up for tumor regrowth, and we have planned adjuvant radiotherapy in the event of a tumor recurrence. The patient maintains a good condition at 2 years’ follow-up. Histopathology confirmed the diagnosis of adamantinomatous craniopharyngioma.

These are the references and thanks for watching.

## Disclosures

The authors report no conflict of interest concerning the materials or methods used in this study or the findings specified in this publication.

## Author Contributions

Primary surgeon: Das. Assistant surgeon: Kanuru, Bhaisora. Editing and drafting the video and abstract: Das. Critically revising the work: Das, Nigam, Bhaisora. Reviewed submitted version of the work: Das, Rai. Approved the final version of the work on behalf of all authors: Das. Supervision: Das, Srivastava. Pathology: Nigam.

## Supplemental Information

### Patient Informed Consent

The necessary patient informed consent was obtained in this study.
